# A Unique Electrical Thermal Stimulation System Comparable to Moxibustion of Subcutaneous Tissue

**DOI:** 10.1155/2014/518313

**Published:** 2014-07-13

**Authors:** Hyoun-Seok Myoung, Kyoung-Joung Lee

**Affiliations:** Department of Biomedical Engineering, Yonsei University, 234 Maeji, Heungeop, Wonju, Gangwon-do 220-842, Republic of Korea

## Abstract

Moxibustion strengthens immunity and it is an effective treatment modality, but, depending on the material quantity, shape, and composition, the thermal strength and intensity can be difficult to control, which may cause pain or epidermal burns. To overcome these limitations, a heat stimulating system which is able to control the thermal intensity was developed. The temperature distributions on epidermis, at 5 mm and 10 mm of depth, in rabbit femoral tissue were compared between moxibustion and the electric thermal stimulation system. The stimulation system consists of a high radio frequency dielectric heating equipment (2 MHz frequency, maximum power 200 W), isolation probe, isolation plate, negative pressure generator, and a temperature assessment system. The temperature was modulated by controlling the stimulation pulse duty ratio, repetition number, and output. There were 95% and 91% temperature distribution correlations between moxibustion and the thermal stimulus at 5 mm and 10 mm of depth in tissue, respectively. Moreover, the epidermal temperature in thermal stimulation was lower than that in moxibustion. These results showed that heat loss by the electric thermal stimulation system is less than that by the traditional moxibustion method. Furthermore, the proposed electric thermal stimulation did not cause adverse effects, such as suppuration or blisters, and also provided subcutaneous stimulation comparable to moxibustion.

## 1. Introduction

Complementary and alternative medicine has newly emerged among healthy individuals as well as patients in recent years. This treatment includes adjuvant, subcultural, and unorthodox therapies, along with prevention, diagnosis, and treatment outside of traditional western medicine [1]. Among the available alternative therapies, acupuncture and other eastern medicines are important components of disease prevention and treatment. Numerous clinical and pathologic studies have been performed in Korea as well as other countries and have been applied in the treatment of many diseases. However, acupuncture knowledge has progressed primarily owing to clinical experience rather than objective data. Clinical acupuncture studies are lacking; notably, the term acupuncture is virtually unrecognized in eastern medicine. Acupuncture research has focused on experimental studies, and analysis of its clinical effects on the body is lacking. Moxibustion is a traditional eastern treatment that places burning wormwood directly onto the skin or a buffer layer. The heat reportedly stimulates the acupuncture point, accelerates circulation, and enhances immunity.

Current reports show that moxibustion effectively treats poor circulation, chronic urticaria, and chronic cough [2–4], but other studies report unintended adverse effects from the direct stimulation including pain, blistering, and suppuration [5, 6]. These adverse effects are caused by the difficulty in modulating the heat stimulus, which varies according to the material quantity, shape, and composition. Therefore, an easily controlled system minimizing the adverse effects associated with moxibustion is needed.

Previous moxibustion studies only evaluate the specific heat characteristics or placebo effect. Manufactured systems based on currently available data cannot replicate moxibustion but instead provide simple thermal stimulation. For example, high-frequency thermal stimulation provides topical anesthesia during skin treatment and thermal-based tumor removal [7, 8]. However, the currently available method generates intense heat on the skin surface, and the electrode may cause burns; thus, the modality cannot provide heat comparable to that during moxibustion. In a similar study evaluating thermal stimulation as an alternative to moxibustion, the thermal distribution was examined in pig tissue specimens and phantoms. Yet, the study failed to account for vasculature effects and species-specific characteristics and thus its performance in humans is unknown [9].

The present study describes a novel system and protocol with the goal of minimizing the limitations associated with traditional moxibustion, including epidermal burns and poor controllability. An electrical thermal stimulation system targeting the subcutis in anesthetized rabbits was developed and a protocol minimizing heat stimulus was designed to obtain a temperature distribution similar to moxibustion.

## 2. Materials and Methods

### 2.1. Moxibustion

**Figure 1:**
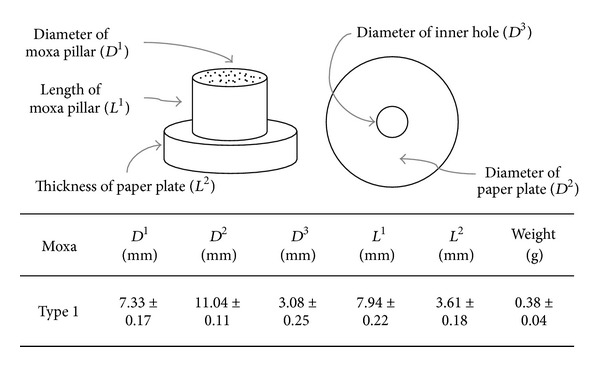
Schematic illustration of the commercial indirect moxibustion apparatus used in the present study. Heat is transferred through the cylindrical moxa pillar (top left). The paper plate (top right) serves as a buffer between the pillar and skin and is similarly cylindrical with a central hole. Moxa pillar and paper buffer dimensions are shown (bottom).

Moxibustion can be performed in several ways; among these are the direct method, which directly contacts the skin, and the indirect method, which places a buffer layer of ginger, garlic, and salt between the skin and apparatus. The direct method must be performed by an expert, but the indirect method can be performed by anyone, and thus is the focus of the present study. The commercial moxibustion apparatus includes an air or paper buffer layer.  Figure 1 illustrates the apparatus shape and size used in this present study. A paper buffer layer was used.

### 2.2. Animal Subjects

Five male New Zealand white rabbits aged 18 months and weighing 3.6 ± 0.2 kg were evaluated. The experiment was performed on the left and right femoral skin five times per side. Subjects were anesthetized using combined tiletamine/zolazepam (Zoletil 0.1 mL/kg) and xylazine (Rompun 0.03 mL/kg) administered intraperitoneally; the fur was removed from the target site, and heat stimulation was applied. All animal use and protocols were approved by the Institutional Animal Care and Use Committee of Yonsei University (IACUC).

### 2.3. Electrical Thermal Stimulation System

**Figure 2:**
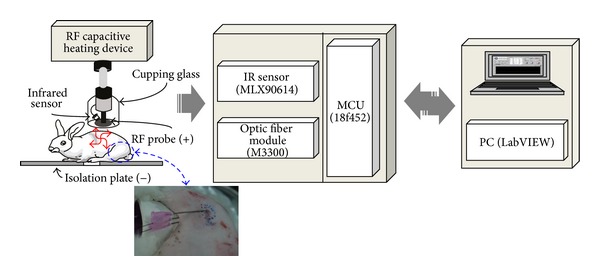
Schematic diagram of the electric high-frequency stimulation system.

The electrical thermal stimulation system was constructed from a clinical high-frequency dielectric heating equipment commercialized for pain relief (Hardville, South Korea). The electrical thermal stimulation generates heat by emitting high-frequency pulses that focus heat energy onto the target tissue. The system comprises a high-frequency dielectric heating equipment, isolation probe, isolation plate, thermometer, and a system control unit; a schematic diagram is shown in Figure 2. The system generated a high dielectric 2 MHz frequency and a maximum 200 W power; an insulating coating minimizes the risk of electric shock. The high frequency was assessed by a medical optical temperature analysis unit (m3300, LumaSense, USA), which determined the subcutaneous temperature distribution. A noncontact infrared sensor (MLX90614, Melexis, Belgium) measured the subcutis and probe temperatures during thermal stimulation. Temperature distribution data were compiled and analyzed using LabVIEW (Ver.8.6, National Instrument, USA) for Windows. 

The subject was placed between the positive probe (+) and negative plate (−), and a stimulation protocol mimicking the moxibustion temperature distribution was applied. A 2 MHz frequency and maximum 200 W power output were applied. To minimize the edge current, the probe was constructed as a cylinder, and the 1.5 cm probe diameter simulated a surface area similar to that in moxibustion. Electric shock was minimized by applying a 0.28 mm polyurethane coating onto the probe and using an isolated plate. During conventional moxibustion, there was a 4 min flame period followed by a 14 min thermal effect; electrical stimulation was applied for 18 min to the target site. During thermal stimulation, the skin surface and probe temperatures were monitored and electric shock was minimized using a noncontact infrared temperature sensor (Figure 2). Thermal skin damage reportedly occurs at 44°C; therefore, to minimize tissue destruction, the electric unit temperature did not exceed 42°C [10, 11].

### 2.4. Temperature Assessment

**Figure 3:**
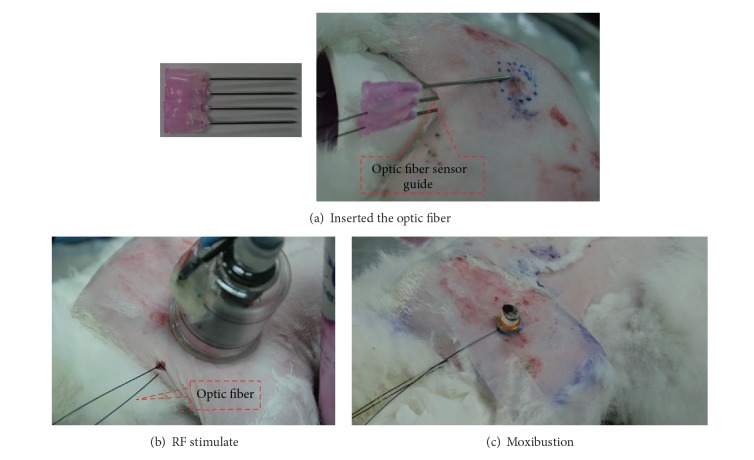
Constructed thermal stimulation and temperature assessment during electric thermal stimulation and moxibustion. (a) Insertion of the optic fiber sensor guide, (b) temperature assessment during electrical thermal stimulation, and (c) temperature assessment during moxibustion.

During electrical thermal stimulation, conventional transducers and contact sensors can malfunction due to ambient electrical field and loud noises; thus, assessing temperature in real time is difficult. Therefore, an optical temperature analyzer and noncontact infrared sensor were used to measure temperature distribution during thermal stimulation, summarized in Figure 3. The optical temperature analyzer (Luxtron) is not affected by strong electrical fields and has high repeatability and accuracy. After anesthetic induction, a needle was inserted 5 mm subcutaneously over the femoral region, and the optical temperature probe fiber was inserted into the needle; a fiber sensor guide was then inserted 30 mm subcutaneously (Figure 3(a)). The temperature was measured at the optic fiber endpoint and transmitted through the fiber without outside interference. The thermal probe was attached to the skin using negative pressure applied to the cup using a negative pressure motor (KPV36E, Koge Electronics, Korea) and a solenoid valve maintaining a consistent 10–12 kPa pressure.

As Figure 3 illustrates, the optic fiber sensor guide cannot be inserted at the same anatomic location with each temperature measurement. Therefore, to minimize temperature errors with variation of location, the moxibustion and thermal stimulation temperature distributions were measured consecutively.

## 3. Results

### 3.1. Stimulation Protocol Design

**Figure 4:**
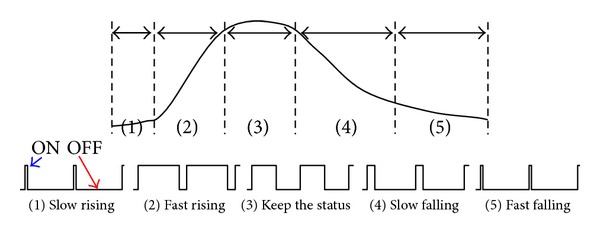
Schematic electric thermal stimulation template protocol. The protocol included ON periods, when high-frequency thermal stimulation was applied, and OFF periods without thermal stimulation; each phase initiates immediately after the previous concludes. The protocol comprised (1) steady temperature increase by shortening the ON period relative to the OFF period, (2) rapid temperature increase by lengthening the ON period, (3) consistent temperature by maintaining equal ON/OFF periods, (4) slow temperature decrease by shortening the ON period relative to the OFF period, and (5) rapid temperature decrease by lengthening the OFF period.

Prior studies showed that, under repetitive electrical stimulations, the temperature increases and decreases rapidly. However, as shown in Figure 4, the temperature during moxibustion peaked rapidly and then decreased slowly. Therefore, a protocol allowing fine temperature control during electrical thermal stimulation was constructed [12]. A temperature distribution mimicking moxibustion was obtained by repetitive electrical stimulations, controlling the ON/OFF ratio and modulating the ON/OFF repetition, summarized in Figure 4.

An electrical thermal protocol was devised from the template illustrated in Figure 4 to stimulate temperature distributions comparable to that of moxibustion, summarized in Table 1. Phases 1 and 2 correspond to the flame initiation during moxibustion, which slowly applies heat and steadily increases the temperature. Phase 3 corresponds to the phase of rapid temperature rise 5 mm subcutaneously during moxibustion. And phase 4 corresponds to the phase of keeping the temperature 5 mm subcutaneously during moxibustion. During phases 1, 2, 3, and 4, the temperature increases over approximately 5 minutes during moxibustion; the electrical thermal stimulation resulted in a temperature distribution comparable to that with moxibustion. Phases 5, 6, 7, 8, and 9 correspond to the moxibustion phase during which the flame dissipates, and the temperature steadily decreases.

### 3.2. Temperature Distributions during Moxibustion and High-Frequency Stimulation

**Figure 5:**
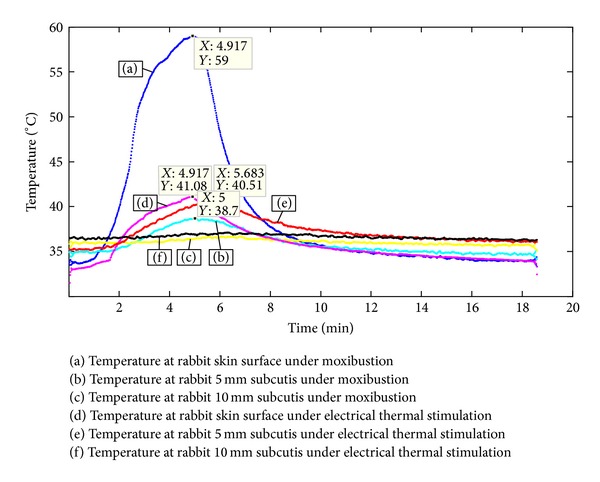
Epidermal and subcutaneous temperature distributions during moxibustion and electric thermal stimulation. The temperature was measured during moxibustion at skin surface (blue), 5 mm subcutis (light blue), and 10 mm subcutis (yellow) overlying the femoral region and during electric thermal stimulation at skin surface (pink), 5 mm subcutis (red), and 10 mm subcutis (black).

Figure 5 compares the temperature distributions in epidermis and subcutis after applying electrical thermal stimulation and moxibustion. Three points (epidermis, 5 mm, and 10 mm in depth) were selected experimentally since the change of temperature rarely occurred at over 10 mm deep during moxibustion in previous study. During moxibustion, the epidermal temperature peaked at 58.41 ± 0.28°C, but the temperature at 5 mm subcutaneously was 39.25 ± 0.55°C, indicating a large heat loss. During electrical thermal stimulation, the maximum epidermal temperature was comparably lower at 39.46 ± 0.29°C and the temperature at 5 mm subcutis was 39.24 ± 0.33°C, indicating minimal heat loss. Similar temperature distributions at 5 mm and 10 mm subcutaneously were observed between moxibustion and electric thermal stimulation, and then the correlation coefficients of *R* = 0.95 and *R* = 0.91 were shown, respectively.

## 4. Discussion and Conclusion

This study sought to address the limitations of moxibustion due to burns and poorly controlled intensity by designing an electrical thermal stimulation system and protocol that can provide an effective thermal subcutaneous stimulus on an anesthetized rabbit. The subcutaneous temperature distributions between the two techniques were comparable, confirmed by the data correlation coefficients (*R* = 0.95 at 5 mm subcutis and *R* = 0.91 at 10 mm subcutis). The infrared hot plate is the only currently available heat stimulation method comparable to moxibustion. The technique focuses heat epidermally, but significant heat is lost subcutaneously [13]. The moxibustion temperature distribution changed significantly between the epidermis and 5 mm subcutis, with a peak temperature change of up to 19.16°C. Comparatively, the electrical stimulation temperature distribution using the protocol summarized in Table 1 showed minimal heat loss of only 0.22°C between the epidermis and 5 mm subcutis. Despite the electrode mechanism targeting high heat epidermally, the unit effectively transmitted heat subcutaneously. Furthermore, the technique avoided the adverse effects such as suppurative blisters typically associated with moxibustion; yet, it also achieved a similar subcutaneous temperature distribution. Unlike the previous studies evaluating thermal heat on pig tissue and phantom specimens, the new system was applied to rabbits, incorporating the vasculature and subcutaneous characteristics; these findings confirm that electric thermal stimulation comparable to traditional moxibustion can be achieved* in vivo*. Also, we can stimulate subcutis at 20 mm thermally by modifying the protocol, which is ensured in our previous study. The presently described clinical protocol has limitations not being applicable to all moxibustion types. However, this study illustrates the potential of electric thermal stimulation to minimize adverse effects and effectively transmit heat subcutaneously. In further research, this electrical stimulation system should be compared with moxibustion having other types and conditions. Also, we will evaluate the therapeutic effect and the application in human.

## Figures and Tables

**Table 1:** Electrical thermal stimulation protocol mimicking the moxibustion temperature distribution.

Phase	ON (ms)	OFF (ms)	REP
1	5	495	80
2	80	420	100
3	499	1	390
4	450	550	45
5	20	980	125
6	10	990	185
7	5	995	150
8	2	998	157
9	1	999	145

ON, application of high-frequency thermal stimulation; OFF, absent stimulation; REP, pulse repetition number. Table 1 shows a protocol for obtaining temperature distribution similar to that of moxibustion at the depth of 5 mm in subcutaneous tissue.
